# Safety and immunogenicity of the third and fourth doses of vaccine against SARS-CoV-2 following a 2-dose regimen of inactivated whole-virion SARS-CoV-2 vaccine

**DOI:** 10.1038/s41598-023-45735-7

**Published:** 2023-11-13

**Authors:** Romanee Chaiwarith, Poramed Winichakoon, Parichat Salee, Tavitiya Sudjaritruk, Jiraprapa Wipasa, Kriangkrai Chawansuntati, Saowaluck Yasri, Harit Thongwitokomarn, Kawisara Krasaewes, Sethawut Ruangsirinusorn, Jutarat Praparattanapan, Nuttarika Solai, Khanuengnit Nuket, Darakorn Boonmee, Orapin Chaichana, Oramai Mueangmo, Jutamad Saheng, Worawan Wongjak

**Affiliations:** 1 Division of Infectious Diseases and Tropical Medicine, Department of Internal Medicine, Faculty of Medicine, Chiang Mai University, Chiang Mai, 50200 Thailand; 2 Division of Infectious Diseases, Department of Pediatrics, Faculty of Medicine, Chiang Mai University, Chiang Mai, 50200 Thailand; 3 Clinical and Molecular Epidemiology of Emerging and Re-Emerging Infectious Diseases Research Cluster, Faculty of Medicine, Chiang Mai University, Chiang Mai, 50200 Thailand; 4 Research Institute for Health Sciences, Chiang Mai University, Chiang Mai, 50200 Thailand

**Keywords:** Infectious diseases, Infectious diseases

## Abstract

This study followed healthcare personnel (HCP) who had completed a primary series of CoronaVac and then received the third and fourth doses of COVID-19 vaccine. The primary objective was to determine the seroconversion rate of neutralizing antibodies against wild-type SARS-CoV-2 and VOCs at day 28 after the third dose of vaccine and day 28 after the fourth dose of vaccine. This prospective cohort study was conducted at Maharaj Nakorn Chiang Mai Hospital, a tertiary care hospital affiliated to Chiang Mai University from July 2021 to February 2022. Two hundred and eighty-three participants were assessed for eligibility; 142 had received AZD1222 and 141 BNT162b2 as the third dose. Seroconversion rates using a 30% inhibition cutoff value against wild-type SARS-CoV-2 were 57.2%, 98.6%, 97.8%, and 98.9% at points before and after the third dose, before and after the fourth dose, respectively among those receiving AZD1222 as the third dose. Frequencies were 31.9%, 99.3%, 98.9%, and 100% among those receiving BNT162b2 as the third dose, respectively. The seroconversion rates against B.1.1.529 [Omicron] were 76.1% and 90.2% (p-value 0.010) at 4 weeks after the third dose in those receiving AZD1222 and BNT162b2 as the third dose, respectively. After a booster with the mRNA vaccine, the seroconversion rates increased from 21.7 to 91.3% and from 30.4 to 91.3% in those receiving AZD1222 and BNT162b2 as the third dose, respectively. No serious safety concerns were found in this study. In conclusion, antibody responses waned over time regardless of the vaccine regimen. The booster dose of the vaccine elicited a humoral immune response against SARS-CoV-2 including SARS-CoV-2 variants of concern, including B.1.1.529 [Omicron], which was circulating during the study period. However, the results might not be extrapolated to other Omicron sublineages.

## Introduction

Different vaccine platforms against COVID-19 have been developed after the pandemic, and the most commonly available for use include mRNA vaccines (e.g. BNT162b2, mRNA1273), viral vector vaccines (AZD1222, Ad26.COV2-S), and whole-cell inactivated virus vaccines (CoronaVac, COVID-19 vaccine BIBP)^1^. A vaccine elicits both humoral and cellular immune responses; which work synergistically to protect against SARS-CoV-2 infection^2,3^. Detection of the humoral response via serological testing is the most feasible method of evaluating immunogenicity, and it has been acknowledged that the levels of neutralizing antibody tend to be an acceptable predictor of vaccine efficacy^4^. Higher neutralizing antibody levels against wide-type SARS-CoV-2 were detected in those receiving homologous prime-boost mRNA vaccine than following the other homologous vaccine platforms^5–7^. Heterologous prime-boost strategies compared with the homologous approach showed different results depending on the type of prime-boost vaccine. Prime-boost with AZD1222/BNT162b2 resulted in higher antibody levels than homologous AZD1222, whereas BNT162b2/AZD1222 showed lower antibody levels than homologous BNT162b2, and BNT162b2/mRNA1273 elicited a higher antibody response than homologous BNT162b2^6^. Therefore, a heterologous regimen boosted by mRNA elicited a more effective immune response than boosting by other vaccine types, particularly with mRNA1273^8,9^. As waning antibody levels occurred, and variants of concern (VOCs) emerged, booster dosing after a homologous or heterologous vaccine schedule is recommended to maintain high antibody levels^6,10–12^. In Thailand, at the beginning of the pandemic, healthcare personnel (HCP) were prioritized for vaccination and the first available COVID-19 vaccine was a whole cell inactivated virus vaccine (CoronaVac). The primary series of CoronaVac included a 2-dose regimen. Later, AZD1222 followed by BNT162b2 became available for HCP working in the public health sector, HCP could elect to receive either AZD1222 or BNT162b2 for the third dose. When mRNA1273 was available later on, HCP could elect to receive either BNT161b2 or mRNA1273 as a fourth dose. Whether to receive the booster doses and to select the type of booster vaccine were based upon HCP’s own decision. The study of safety and immunogenicity of the third dose of AZD1222 or BNT162b2 following the final dose of the primary series of CoronaVac resulted in a higher antibody level in those who received the mRNA vaccine^11,13,14^. This study tracked HCP who completed a primary series of CoronaVac and received a third and fourth dose of COVID-19 vaccine. The primary objective was to determine the seroconversion rate of neutralizing antibodies against wild-type SARS-CoV-2 and VOCs at day 28 after the third dose of vaccine and day 28 after the fourth dose of vaccine. The secondary objectives were: (1) to determine the % inhibition of neutralizing antibodies to wild-type SARS-CoV-2 and VOCs following the third and the fourth doses of vaccine, and (2) to determine the binding antibodies following the third and the fourth doses of vaccine.

## Results

**Figure 1:**
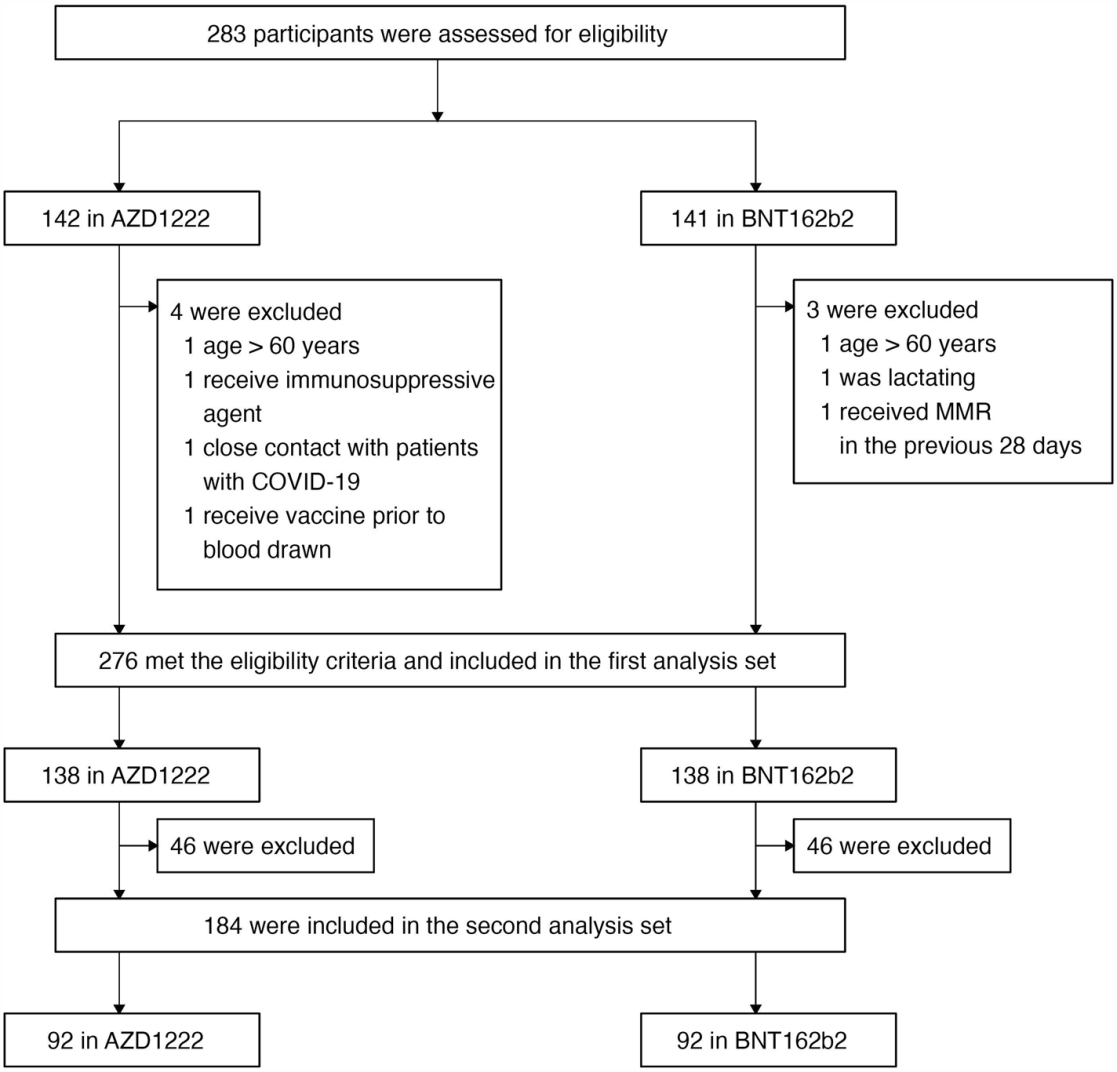
The flow of the study participants enrolled into this study.

Two hundred and eighty-three were assessed for eligibility; 142 had received AZD1222 (AstraZeneca-Oxford) and 141 received BNT162b2 (Pfizer-BioNTech) as the third dose. A total of 276 were included in the first set and 184 were included in the second set of analyses. (Fig. 1) Participants who received AZD1222 were older and more likely to have underlying diseases compared with those who received BNT162b2 as a third dose. There were no participants receiving immunosuppressive/ immunomodulatory agents. Four participants who had malignancies were in remission at the time of enrollment. The median duration between the second dose of CoronaVac (Sinovac-CoronaVac) and the third dose was 68 days (IQR 51, 110) for those receiving AZD1222 and was 71 days (IQR 65, 130) for those receiving BNT162b2 (p-value = 0.007). The median duration between the third and the fourth dose of vaccine was 155 days (IQR 154, 155) for those receiving mRNA1273 (Moderna-NIH) followed by AZD1222 and was 147 days (IQR 147, 148) for those receiving mRNA vaccine followed by BNT162b2 (p-value < 0.001). Among the 92 participants receiving mRNA as the fourth dose, 76 received BNT162b2 and 16 received mRNA. There were imbalances between the characteristics between the 2 vaccine schedule groups as shown in Table 1.

**Table 1:** Characteristics of participants before the third dose and the fourth dose.

Characteristics before the third dose	AZD1222 (N = 138)	BNT162b2 (N = 138)	p-values
Median (IQR), age (years)	50.4 (44.5, 56.1)	30.7 (27.3, 42.7)	< 0.001
Female	108 (78.3)	90 (65.2)	0.016
Occupation			
Nurse	84 (60.9)	55 (39.9)	< 0.001
Physician	18 (13.0)	50 (36.2)	
Others	36 (26.1)	33 (23.9)	
Underlying diseases			
Dyslipidemia	31 (22.5)	9 (6.5)	< 0.001
Hypertension	21 (15.2)	9 (6.5)	0.020
Asthma	7 (5.1)	13 (9.4)	0.164
Diabetes mellitus	7 (5.1)	3 (2.2)	0.335
Thyroid diseases	4 (2.9)	2 (1.4)	0.684
Malignancy	3 (2.2)	1 (0.7)	0.622
Median (IQR) body mass index (kg/m^2^)	23.3 (21.5, 25.9)	22 (20.0, 25.2)	0.008
Median (IQR) duration from the second dose to the third dose (days)	68 (51.0, 110.0)	71 (65.0, 130.0)	0.007
Participants in whom duration from the second dose to the third dose > 90 days	48 (34.8)	48 (34.8)	1.000

Characteristics before the fourth dose	AZD1222 (N = 92)	BNT162b2 (N = 92)	p-values
Median (IQR), age (years)	51.8 (46.0, 56.7)	32.2 (27.6, 43.3)	< 0.001
Female	78 (84.8)	62 (67.4)	0.006
Occupation			
Nurse	59 (64.1)	40 (43.5)	< 0.001
Physician	11 (12.0)	30 (32.6)	
Others	22 (23.9)	23 (25.0)	
Underlying diseases			
Dyslipidemia	23 (25.0)	8 (8.7)	0.003
Hypertension	15 (16.3)	7 (7.6)	0.069
Asthma	4 (4.3)	11 (12.0)	0.059
Diabetes mellitus	6 (6.5)	2 (2.2)	0.278
Thyroid diseases	3 (3.3)	1 (1.1)	0.621
Malignancy	1 (1.1)	1 (1.1)	1.000
Median (IQR) body mass index (kg/m^2^)	23.3 (21.5, 25.9)	22.1 (19.9, 24.7)	0.006
Median (IQR) duration from the third dose to the fourth dose (days)	147.0 (147.0, 148.0)	155 (154.0, 155.0)	< 0.001
Participants in whom duration from the third dose to the fourth dose > 90 days	31 (33.7)	35 (38.0)	0.539
Type of the fourth dose vaccine			
mRNA-1273	92 (100.0)	16 (17.4)	< 0.001

Categorical data are presented in number (%).

### RBD-specific-binding antibody against wild-type (WT) SARS-CoV-2

**Figure 2:**
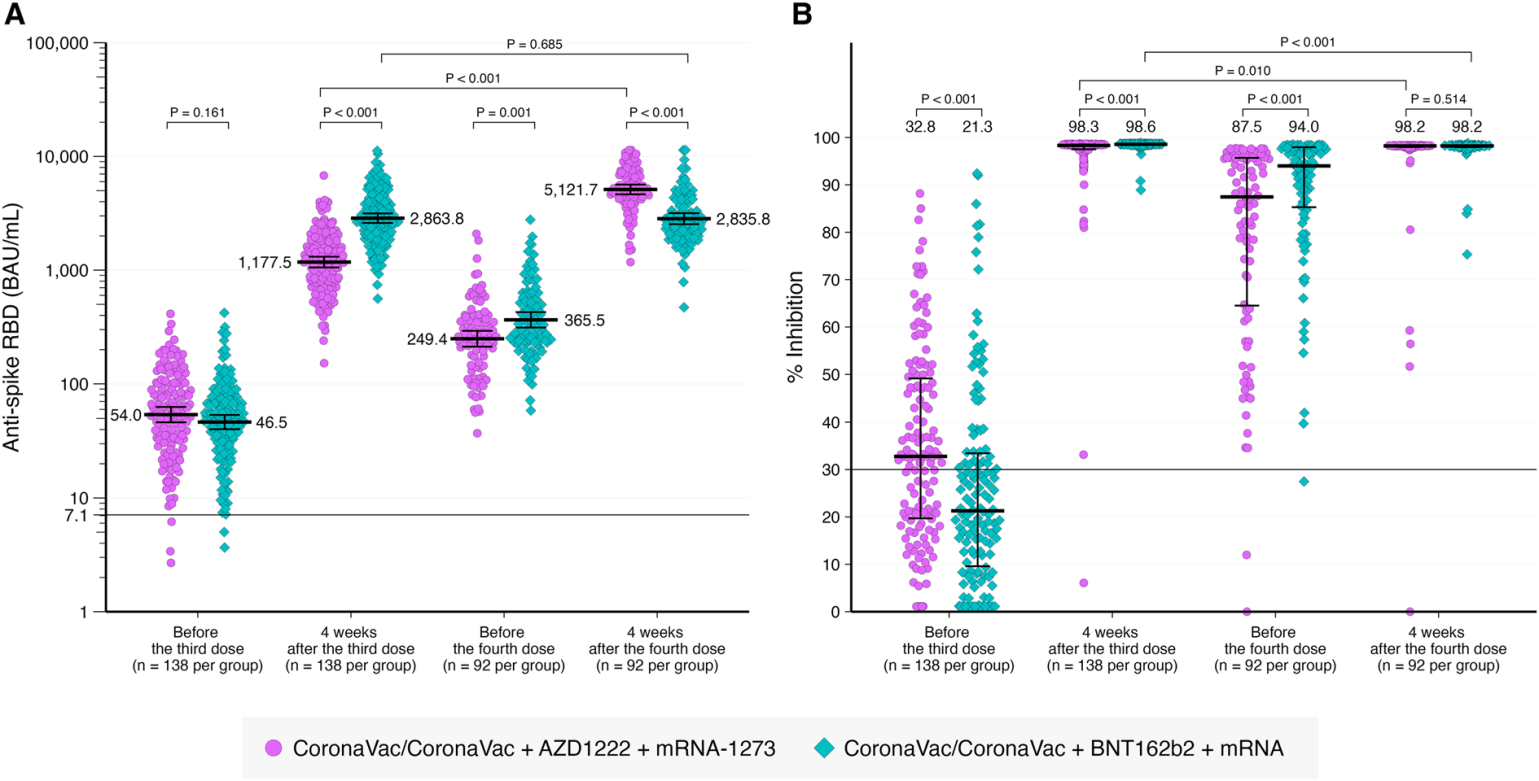
RBD-specific IgG geometric mean titer (GMT) (**A**) and % inhibition against (**B**) wild-type SARS-CoV-2 at different time points.

The GMT of RBD-specific-binding IgG level were 54 BAU/mL and 46.5 BAU/mL for those receiving AZD1222 and BNT162b2, respectively (p = 0.161) before receiving the third dose of vaccine (Table 2 and Fig. 2). The RBD-specific IgG level increased at 28 days after the third dose, declined before the fourth dose, and increased at 28 days after the fourth dose as shown in Fig. 2. A higher antibody level was observed in those receiving BNT162b2 as a third dose compared to those receiving AZD1222 at 28 days after the third dose and before the fourth dose. After the fourth dose with mRNA vaccine, the antibody level was higher among those receiving AZD1222 as a third dose in comparison to those receiving BNT162b2. Participants receiving BNT162b2 as the third dose followed by mRNA1273 had a higher GMT than those receiving BNT162b2 followed by BNT162b2. (3,700.4 v.s. 2679.3, p-value = 0.028).

**Table 2:** RBD-specific antibodies, % inhibition, and seroconversion rate against WT-SARS-CoV-2.

Time point	Anti-spike RBD (BAU/mL) (median, IQR)		P-value	% Inhibition (median, IQR)		P-value	Seroconversion rate (n, %)		P-value
	AZD1222	BNT162b2		AZD1222	BNT162b2		AZD1222	BNT162b2	
Overall									
Before the 3rd dose	54.0 (46.3, 63.1)	46.5 (40.2, 53.7)	0.161	32.8 (19.6, 49.5)	21.3 (9.3, 33.7)	< 0.001	79 (57.2)	44 (31.9)	< 0.001
4-week after the 3rd dose	1177.5 (1056.1, 1312.8)	2863.8 (2600.9, 3153.1)	< 0.001	98.3 (97.5, 98.5)	98.6 (98.4, 98.6)	< 0.001	136 (98.6)	137 (99.3)	1.000
Before the 4th dose	249.4 (212.5, 292.7)	365.5 (312.7, 427.2)	0.001	87.5 (64.3, 95.7)	94.0 (85.1, 98.0)	< 0.001	90 (97.8)	91 (98.9)	1.000
4-week after the 4th dose	5121.7 (4640.1, 5653.3)	2835.8 (2536.5, 3170.6)	< 0.001	98.2 (98.0, 98.3)	98.2 (97.9, 98.3)	0.514	89 (98.9)	91 (100.0)	0.497
Duration from the 2nd dose ≤ 90 days									
Before the 3rd dose	80.0 (68.6, 93.4)	67.5 (59.2, 77.1)	0.987	38.0 (23.5, 52.7)	28.4 (17.7, 44.7)	0.008	61 (67.8)	40 (44.4)	0.002
4-week after the 3rd dose	1131.4 (997.3, 1283.5)	2644.2 (2362.5, 2959.6)	< 0.001	98.3 (97.4, 98.5)	98.6 (98.4, 98.6)	< 0.001	90 (100.0)	90 (100.0)	-
Before the 4th dose	224.9 (186.5, 271.2)	300.4 (254.2, 355.1)	0.023	86.4 (61.2, 95.7)	91.9 (83.0, 96.6)	0.018	60 (98.4)	56 (98.2)	1.000
4-week after the 4th dose	5097.7 (4496.4, 5779.3)	2857.4 (2438.8, 3347.9)	< 0.001	98.2 (97.9, 98.3)	98.2 (98.0, 98.3)	0.362	60 (100.0)	57 (100.0)	-
Duration from the 2nd dose > 90 days									
Before the 3rd dose	25.9 (20.6, 32.5)	23.1 (18.3, 29.0)	0.474	22.2 (17.5, 34.2)	8.2 (1.1, 19.2)	<0.001	18 (37.5)	4 (8.3)	0.002
4-week after the 3rd dose	1269.1 (1028.9, 1565.3)	3325.7 (2787.1, 3968.4)	< 0.001	98.4 (97.7, 98.6)	98.6 (98.5, 98.6)	<0.001	47 (97.9)	48 (100.0)	1.000
Before the 4th dose	305.6 (225.9, 413.5)	502.9 (377.5, 670.0)	0.018	88.5 (70.5, 95.7)	97.6 (90.2, 98.2)	0.001	30 (96.8)	35 (100.0)	0.470
4-week after the 4th dose	5170.1 (4371.4, 6114.8)	2800.1 (2417.2, 3243.6)	< 0.001	98.2 (98.1, 98.3)	98.2 (97.8, 98.3)	0.904	29 (96.7)	34 (100.0)	0.469

The number of participants for AZD1222 was 138 before and after the third dose, 92 before the fourth dose and 90 after the fourth dose. The number of participants for BNT162b2 was 138 before and after the third doses, 92 before the fourth dose and 91 after the fourth dose.

### Neutralizing antibody against WT SARS-CoV-2

To compare the level of neutralizing antibody across 4 visits, the % inhibitions performed by the SARS-CoV-2 NeutraLISA were converted to % inhibitions on the in-house sVNT scale^15^. Median (IQR) % inhibitions against wild-type SARS-CoV-2 were 32.8 (19.6, 49.5), and 98.3 (97.5, 98.5) at before and after the third dose, respectively among those receiving AZD1222 as the third dose. The numbers were 21.3 (9.3, 33.7) and 98.6 (98.4, 98.6) at before and after the third dose, respectively among those receiving BNT162b2 as the third dose. (Fig. 2).

All participants received mRNA vaccine as a fourth dose. One hundred percent of those receiving AZD1222 as a third dose, and 82.6% of those receiving BNT162b2 as a third dose received mRNA1273. Median (IQR) % inhibition before and after the fourth dose were 87.5 (64.3, 95.7) and 98.2 (98.0, 98.3), respectively in those receiving AZD1222 as a third dose and were 94.0 (85.1, 98.0) and 98.2 (97.9, 98.3), respectively in those receiving BNT162b2 as a third dose (Table 2).

Percentage inhibitions were higher in those receiving BNT162b2 as a third dose than those receiving AZD1222, except that % inhibition was no different after the fourth dose of vaccine.

### Seroconversion rate against WT-SARS-CoV-2

Seroconversion rates using a 30% inhibition cutoff value against wild-type SARS-CoV-2 were 57.2%, 98.6%, 97.8%, and 98.9% at before and after the third dose, and before and after the fourth dose, respectively among those receiving AZD1222 as the third dose. Frequencies were 31.9%, 99.3%, 98.9%, and 100% among those receiving BNT162b2 as the third dose, respectively (Table 2).

### Neutralizing antibody against SARS-CoV-2 variants of concern

**Figure 3:**
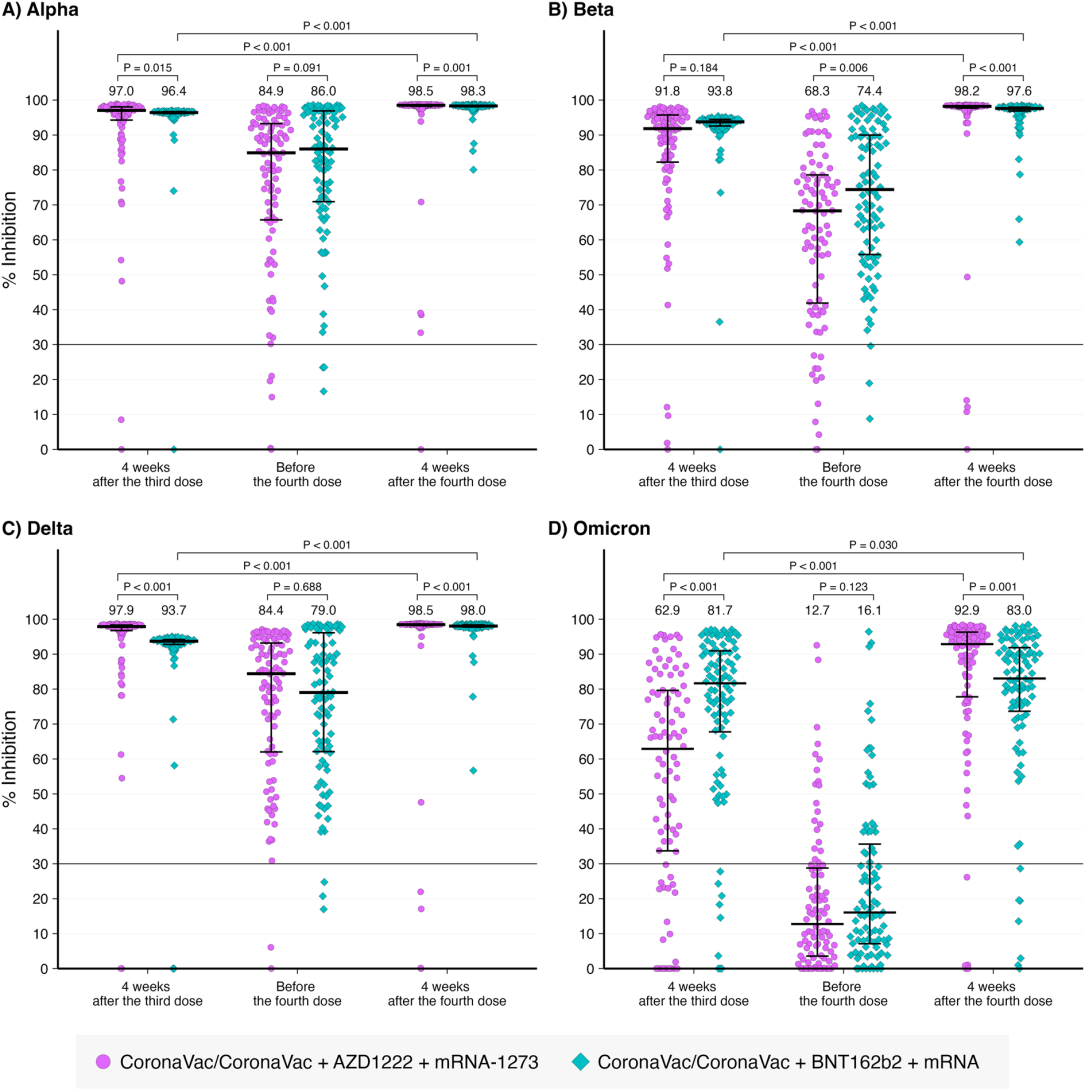
% inhibition against SARS-CoV-2 variants of concern at different time points.

Percentage inhibitions against SARS-CoV-2 VOCs including B.1.1.7 [Alpha], B.1.351 [Beta], B.1.617.2 [Delta], or B.1.1.529 [Omicron] variants are shown in Fig. 3A–D, respectively. The median trends of % inhibition were similar to those of wild-type SARS-CoV-2 in which they were < 30% before the third dose, increased after the third dose, declined before the fourth, and increased after the fourth dose. The median % inhibition against B.1.1.529 declined below 30% before the fourth dose of vaccine.

Comparisons between the 2 vaccine schedules showed that those receiving AZD1222 as the third dose had significantly higher levels of % inhibition at 4 weeks after the third dose against wild-type, B.1.1.7 [Alpha], B.1.617.2 [Delta], but were significant lower against B.1.1.529 [Omicron]. Before the fourth dose, the level of % inhibition was significantly lower against wild-type, B.1.1.7 [Alpha], and B.1.351 [Beta]. After the fourth dose, the % inhibition level against B.1.1.529 [Omicron] was significantly lower in those receiving mRNA following BNT162b2 in comparison with those receiving mRNA1273 following AZD1222.

### Seroconversion rate against SARS-CoV-2 variants of concern

Seroconversion rates using a 30% inhibition cutoff value against SARS-CoV-2 VOCs are shown in Table 3. The seroconversion rate was lowest against B.1.1.529 [Omicron] particularly before the 4th dose, and no difference was detected between those receiving either AZD1222 or BNT162b2 as a third dose. However, after the 4th dose with mRNA vaccine, the seroconversion rate increased to above 90%, regardless of SARS-CoV-2 VOCs and the type of the third dose of vaccine (Table 3).

**Table 3:** % inhibition and seroconversion rate against SARS-CoV-2 variant of concerns.

Time point	% Inhibition (median, IQR)		P-value	Seroconversion rate (n, %)		P-value
	AZD1222	BNT162b2		AZD1222	BNT162b2	
B.1.1.7 [Alpha]						
4-week after the 3rd dose	97.0 (94.1, 98.1)	96.4 (96.1, 96.7)	0.015	90 (97.8)	91 (98.9)	1.000
Before the 4th dose	84.9 (65.7, 93.3)	86.0 (70.9, 96.9)	0.091	87 (94.6)	89 (96.7)	0.720
4-week after the 4th dose	98.5 (98.2, 98.6)	98.3 (98.1, 98.5)	0.001	89 (98.9)	91 (100.0)	0.497
B.1.351 [Beta]						
4-week after the 3rd dose	91.8 (81.9, 95.8)	93.8 (92.6, 94.5)	0.184	88 (95.7)	91 (98.9)	0.368
Before the 4th dose	68.3 (41.6, 78.6)	74.4 (55.7, 90.1)	0.006	80 (87.0)	89 (96.7)	0.028
4-week after the 4th dose	98.2 (97.7, 98.4)	97.6 (96.8, 98.0)	< 0.001	86 (95.6)	91 (100.0)	0.059
B.1.617.2 [Delta]						
4-week after the 3rd dose	97.9 (96.8, 98.3)	93.7 (92.8, 94.2)	< 0.001	90 (97.8)	90 (97.8)	1.000
Before the 4th dose	84.4 (61.8, 93.3)	79.0 (62.0, 96.3)	0.688	90 (97.8)	89 (96.7)	1.000
4-week after the 4th dose	98.5 (98.2, 98.6)	98.0 (97.7, 98.4)	< 0.001	86 (95.6)	91 (100.0)	0.059
B.1.1.529 [Omicron]						
4-week after the 3rd dose	62.9 (33.6, 79.6)	81.7 (67.3, 91.1)	< 0.001	70 (76.1)	83 (90.2)	0.010
Before the 4th dose	12.7 (3.5, 28.8)	16.1 (7.0, 36.8)	0.123	20 (21.7)	28 (30.4)	0.179
4-week after the 4th dose	92.9 (77.6, 96.3)	83.0 (72.7, 92.0)	0.001	84 (93.3)	84 (92.3)	0.789

The number of participants for AZD1222 was 92 after the third dose and before the fourth dose, and 90 after the fourth dose. The number of participants for BNT162b2 was 92 after the third dose and before the fourth dose, and 91 after the fourth dose.

### Subgroup analysis of the second analysis set

Because all participants who received AZD1222 as a third dose received mRNA1273 as a fourth dose and 16 participants (17.4%) of those who received BNT162b2 as a third dose received mRNA1273 as a fourth dose, we performed subgroup analyses to compare antibodies against WT-SARS -CoV-2 and VOCs, and seroconversion rates between subgroups. When comparing participants who received mRNA1273 following AZD1222 with those who received mRNA1273 following BNT162b2, anti-spike RBD levels against WT-SARS -CoV-2 at 4 weeks after the fourth dose were significantly higher in those who received mRNA1273 following AZD1222, although percent inhibition and seroconversion rates were similar. In addition, percent inhibition against SARS-CoV-2 VOCs at 4 weeks after the fourth dose were significantly higher in those who received mRNA1273 following AZD1222 for B.1.351 [Beta] and B.1.1.529 [Omicron] (Tables S1 and S2).

When comparing participants who received BNT162b2 following BNT162b2 and those who received mRNA1273 following BNT162b2, higher levels of anti-spike RBD were observed in participants who received mRNA1273 following BNT162b2 than those who received BNT162b2 following BNT162b2 at 4 weeks after the fourth dose, although percent inhibition and seroconversion rates against WT-SARS-CoV-2 were similar. In addition, the percent inhibition and seroconversion rates against SARS-CoV-2 VOCs in both groups were similar. However, the number of samples in the subgroup analyses was small. The results are shown in the supplemental table (Table S3 and S4).

### The safety endpoint

**Figure 4:**
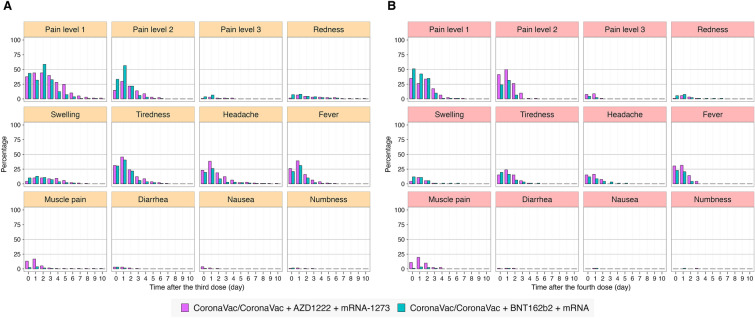
The percentages of local and systemic adverse events following the third (**A**) and the fourth (**B**) dose of vaccine against SARS-CoV-2.

The side effects following the third and the fourth dose of vaccine are shown in Fig. 4A,B. The most common adverse effect was pain at the injection site.

## Discussion

This prospective study confirmed that antibodies waned over time after vaccination. The antibodies against WT-SARS-CoV-2 declined after completion of the primary series of CoronaVac and increased after the third and the fourth dose of COVID-19 vaccine. RBD-specific antibodies against WT-SARS-CoV-2 demonstrated higher levels in those receiving BNT162b2 as the third dose of vaccine than those receiving AZD1222. Previous studies have shown that an mRNA vaccine elicited antibody responses more effectively than AZD1222^6,16^. The highest antibody responses were elicited in participants in whom the primary vaccine series was BNT162b2/BNT162b2 who received mRNA1273, whereas those who received a primary vaccine series with AZD1222/AZD1222 and received mRNA1273 as a third dose had higher antibody responses than those receiving AZD1222 as a third dose^6^. A study in Thailand among those in whom the primary vaccine series was CoronaVac/CoronaVac, the antibody responses were higher elicited in those receiving BNT162b2 than those receiving AZD1222 as a third dose^12^. This study confirmed those results. Previous studies have shown that heterologous prime-boost with an mRNA vaccine as the booster elicited a greater response regarding RBD-specific antibodies and neutralizing antibodies than a homologous prime-boost^6,8,9,17,18^. However, studies in a Thai population showed that homologous BNT162b2/BNT162b2 elicited antibody responses higher than AZD1222/BNT162b2^17,18^. However, after the fourth dose, those receiving mRNA following AZD1222 had a higher level of RBD-specific antibodies than those receiving mRNA following BNT162b2, but this was not the case regarding neutralizing antibodies. Neutralizing antibodies at 4 weeks after the fourth dose was not different between groups against WT-SARS-CoV-2.

In the case of SARS-CoV2 VOCs, after the third dose, the neutralizing antibodies were lowest against B.1.1.529 [Omicron], as described in other reports^12,17,19,20^. The seroconversion rates were similar between groups against SARS-CoV-2 VOCs, except for B.1.1.529 [Omicron] in which those receiving AZD1222 as a third dose showed a lower seroconversion rate than those receiving BNT162b2 as a third dose. The third dose of vaccine elicited neutralizing antibodies against B.1.617.2 [Delta] during the outbreak in Thailand. The antibodies significantly declined by 5 months after the third dose or before the 4th dose against B.1.1.529 [Omicron] with a seroconversion rate of only 20–30%. After the fourth dose, the seroconversion rates were similar between groups for all tested VOCs of SARS-CoV-2. The neutralizing antibodies against B.1.1.529 [Omicron] were significantly lower among those receiving mRNA following BNT162b2 as a third dose compared to those receiving mRNA1273 following AZD1222 as a third dose, although the former group was younger and had a longer interval between vaccine doses. The reason for these results is uncertain. The booster dose elicited an immune response against B.1.1.529 [Omicron], the most common variant during that period.

However, as the neutralizing antibodies waned overtime and there are currently omicron subvariants with increased escape neutralizing antibodies either induced by infection or vaccination^21–23^. Therefore, a monovalent vaccine which contains the spike protein of WT-SARS-CoV-2 may not be sufficient to boost the humoral immune response to neutralize the SARS-CoV-2 variants of concern^24^. Currently, a bivalent vaccine containing the spike protein from WT-SARS-CoV-2 and from the omicron BA.4 and B1.5 sublineages has been recommended to replace monovalent vaccine^25^. Studies comparing those receiving the bivalent vaccine to those who did not received the bivalent vaccine demonstrated a lower hospitalization rate and reduced severity of disease^26^. This was also the case when comparing those receiving the bivalent vaccine with those receiving the monovalent vaccine^24^. Many countries are recommending encouragement of people who are at risk of severe COVID-19 disease to take a booster dose with the vaccines available in those countries^27^.

No serious safety concerns were discovered in this study population, the most common adverse effect was pain at injection site.

This study has several limitations. First, antibody response but not cell-mediated immune response was tested. Studies have shown that even if antibodies did not adequately neutralized the virus, the cell-mediated immune response was still preserved^28^. Therefore, the results must be interpreted with caution. Second, as this is not a randomized controlled trial, participants made their own decision regarding whether to receive either AZD1222 or BNT162b2 as a third dose. Participants who elected to receive AZD1222 were older and therefore were more likely to have underlying diseases compared with those who received BNT162b2 as a third dose. However, all participants in this study were aged 18–59 years and the antibodies were significantly higher after the fourth dose in those who received AZD1222 as a third dose. Therefore, the age differences in this study may not have affected the antibody response. In addition, as the vaccination schedules were determined by the hospital administrative, taking into account for the availability of personnel for mass vaccination; therefore, the durations between the second and the third doses and between the third and the fourth doses were different in the 2 groups. The longer lag time between the last dose of the primary vaccine series and the booster dose correlated with better humoral immune response than the shorter one^29^. However, the difference found in this study may not be clinically significant as they were only 1 week apart. Third, due to the precision of the percentage inhibition of the neutralizing antibodies, even if the results were statistically significant, they may not be clinically significant e.g. 98.6% v.s. 98.3% (p-value < 0.001) at 4 weeks after the third dose against WT-SARS-CoV-2.

In conclusion, the study in HCP without explicit immunocompromised status demonstrated that antibody responses waned overtime regardless of vaccine regimen. The booster dose of the vaccine elicited a humoral immune response against SARS-CoV-2 including SARS-CoV-2 VOCs including B.1.1.529 [Omicron], which was circulating during the study period. However, the results might not be extrapolated to other Omicron sublineages.

## Methods

This prospective cohort study was conducted at Maharaj Nakorn Chiang Mai Hospital, a tertiary care hospital affiliated to Chiang Mai University from July 2021 to February 2022. The inclusion criteria were healthcare personnel who were: (1) aged 18–59 years, (2) had received the second dose of CoronaVac at least 4 weeks before enrollment, (3) had received a third (booster) dose of vaccine against SARS-CoV-2 of either the Oxford–AstraZeneca chimpanzee adenovirus vectored vaccine (ChAdOx1 nCoV-19; AZD1222) or mRNA vaccine (BNT162b2) at Maharaj Nakorn Chiang Mai Hospital, (4) received a fourth (booster) dose of vaccine against SARS-CoV-2 of either BNT162b2 or mRNA1273 at Maharaj Nakorn Chiang Mai Hospital, (5) were able to adhere to the follow-up visit, and (6) provided informed consent. Participants were excluded if they: (1) were previously diagnosed with COVID-19 in the past 90 days before enrollment and during the study period, (2) had a high-risk epidemiology history within 14 days before enrolment e.g. close contact with index cases or visiting/ living in outbreak area, (3) had received vaccines against SARS-CoV-2 other than CoronaVac, (4) were participating in other vaccine clinical trials, (5) were in receipt of blood products, blood components, or immunoglobulin within the past 90 days, (6) were in receipt of attenuated live vaccine within the past 28 days, (7) were in receipt of inactivated or subunit vaccines (other than COVID-19 vaccine) within the past 14 days, (8) were women who were lactating or were planning pregnancy during the study period, (9) had a known allergy to any vaccine component, (10) had signs and symptoms of active skin infection at the injection site, and (11) were receiving immunosuppressive/ immunomodulatory agents at the time of enrollment i.e. glucocorticoids (equivalent to prednisolone ≥ 20 mg/day for at least 3 weeks), methotrexate, cyclophosphamide, cyclosporin, tacrolimus, azathioprine, mycophenolate mofetil, Janus Kinase inhibitors, interleukin-6 inhibitors, and tumor necrosis factor inhibitors.

**Figure 5:**
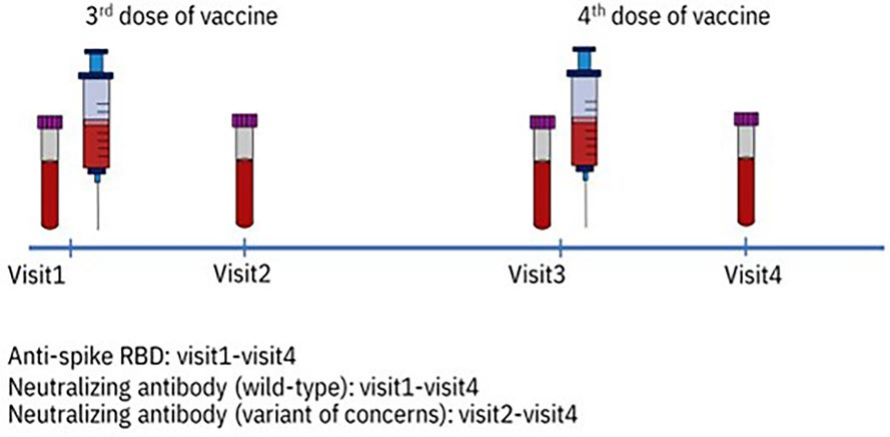
Timeline of blood collection in this study.

Blood (6 ml) was collected at enrollment (before the third dose, visit 1), 4 weeks after the third dose (visit 2), before the fourth dose (visit 3), and 4 weeks after the fourth dose (visit 4). (Fig. 5) Serum was separated to test for: (1) SARS-CoV-2 IgG II Quant assay (Abbott Laboratories Inc, IL, USA) against wild-type virus, and (2) SARS-CoV-2 NeutraLISA (EUROIMMUN Medizinische Labordiagnostika AG, Lübeck, Germany) at visits 1 and 2 or in-house SARS-CoV-2 surrogate virus neutralization test (in-house SARS-CoV-2 sVNT) for visits 2, 3 and 4 were performed against wild-type (WT). In-house sVNT were performed against VOCs of SARS-CoV-2 i.e. Alpha, Beta, Delta, Omicron at visits 2, 3, and 4.

### Statistical analysis

#### Sample size calculation

The sample size was calculated based upon the seroconversion rate at 4 weeks after the third dose of vaccine (visit 2) which was 90%^17^, with an alpha 0.05, and absolute precision of 0.05, and the number of participants required in each arm was 139. In addition, for comparison of the seroconversion rates between those receiving BNT162b2 (95%) and AZD1222 (85%) as a third dose^17^, with an alpha of 0.05 and power 80%, the number of patients required in each group was 138. However, for the analysis for serology against wild-type SARS-CoV-2 and VOCs at before and after the fourth dose of vaccine, only 92 participants in those receiving AZD1222 or BNT162b2 as the third dose were randomly selected for analysis due to budget constraints.

#### Data analysis

The first analytical set included 256 participants receiving the third dose of vaccine. The second set included 184 participants receiving the fourth dose of vaccine. Demographic data are described as number (%), mean ± SD, and median (IQR) as appropriate. Anti-RBD-WT IgG levels were transformed to log base 10 and are presented as geometric mean concentration (GMC) with a 95% confidence interval (95% CI).

Comparisons of demographic data, anti-spike RBD, % inhibition between groups were made using the student’s t-test, Mann–Whitney U test, Chi-square test or Fisher’s exact test. Comparisons of Anti-RBD-WT IgG levels, and % inhibition between time after vaccination were performed using Wilcoxon matched-pairs signed-rank test. A two-sided test with a significance level of p < 0.05 was used to determine statistical significance. All statistical analyses were performed using Stata statistical software version 14 (Stata Statistical Software: Release 14, Stata Corporation, College Station, TX), and all figures were generated using R version 4.1.2.

### Laboratory assays

#### RBD-specific binding antibody

The SARS-CoV-2 IgG II Quant assay (Abbott Laboratories Inc, IL, USA)^30^ was used to test for RBD-specific antibodies. The quantitative antibodies are presented as arbitrary units (AU)/mL and this assay measured the concentration of anti-RBD-WT IgG levels between 21 and 40,000 AU/mL. Those values were converted to binding antibody units (BAU)/ mL by multiplying by 0.142 per WHO recommendations^31^. The cut-off level for a positive result was ≥ 50 AU/mL (7.1 BAU/mL).

#### Neutralizing antibody

##### Neutralizing antibody against wild-type SARS-CoV-2

At visit 1 and 2, neutralizing antibodies were measured using the SARS-CoV-2 NeutraLISA(EUROIMMUN Medizinische Labordiagnostika AG, Lübeck, Germany). The assay was performed following the manufacturer's instruction. The values identified in this study are presented as percentage inhibition and the cut-off level of seroconversion was 35%^32^. However, for cost saving, we developed in-house sVNT, and the technique is described elsewhere^15^. In-house sVNT was performed at visit 2, 3, and 4. Therefore, 92 patients at visit 2 had data pertinent to neutralizing antibodies from both SARS-CoV-2 NeutraLISA and in-house sVNT.

At visit 1, the values from SARS-CoV-2 NeutraLISA were converted to in-house sVNT using the equation: % inhibition from the commercial sVNT = (0. 9968 × % inhibition from in-house sVNT) + 1.3587^15^. At visit 2, % inhibition from in-house sVNT was used for 92 patients. In the case of the other 46 patients who had values only from SARS-CoV-2 NeutraLISA, the conversion to in-house sVNT using the equation above was used for analysis. The cut-off level of seroconversion was 30%^15^.

##### Neutralizing antibody against VOCs

In-house sVNT was modified from the method previously described by Tan et al.^4^. In brief, 50 µL of recombinant angiotensin-converting enzyme 2 (ACE-2) (GenScript, Piscataway, USA) at 4 µg/mL in bicarbonate buffer (pH 9.6) were incubated in 96-well Maxisorp ELISA immunoplates (Thermo scientific, Roskilde, Denmark) at 4 °C overnight. Plates were washed with 0.05% Tween-20 (Calbiochem, Gibbstown, USA) in phosphate buffer saline (PBS) (PBST), followed by blocking with 100 µL 2% bovine serum albumin (PAA Laboratory, Pasching, Austria) in PBS at 37 °C for 1 h. Sera or controls diluted 1:4 in a volume of 60 µL were pre-incubated with equal volumes of 1:200 horse-radish peroxidase (HRP)-conjugated RBD (Wild-type, B.1.1.7 [Alpha], B.1.351 [Beta], B.1.617.2 [Delta], or B.1.1.529 [Omicron] variants) (Genscript, Piscataway, USA) in 96 well U plates for 30 min at 37 °C. After blocking buffer was discarded, 100 µL of the sera-RBD mixture were added to the corresponding wells on ELISA plates. After incubation at 37 °C for 1 h, plates were washed 4 times with PBST and 50 µL of 3, 3′, 5, 5′-tetramethylbenzidine (TMB) substrate solution (Life Technologies, Frederick, USA) were added, and samples were incubated for 30 min. The enzymatic reaction was stopped by adding 50 µL of 0.2 M sulphuric acid. Optical densities were measured at 450 nm on a microtiter plate reader (CLARIOstar®, Ortenberg, Germany).

### Ethical declarations

This study was approved by the Faculty of Medicine Chiang Mai University Ethics Committee number 4, approval number 315/2564. All methods were performed in accordance with relevant guidelines and regulations. Informed consent was obtained from all participants.

### Supplementary Information

Supplementary Table 1.

Supplementary Table 2.

Supplementary Table 3.

Supplementary Table 4.

## Data Availability

The datasets used and/or analysed during the current study available from the corresponding author on reasonable request.
