# Supplementary material for: Safety and immunogenicity of the third and fourth doses of vaccine against SARS-CoV-2 following a 2-dose regimen of inactivated whole-virion SARS-CoV-2 vaccine
Source: Sci Rep. 2023 Nov 13;13:19736. doi: 10.1038/s41598-023-45735-7 (PMC10643552; doi:10.1038/s41598-023-45735-7)
Supplement: Supplementary file 1 — Supplementary Table 1. [file 41598_2023_45735_MOESM1_ESM.pdf]

Table S1. RBD-specific antibodies, % inhibition, and seroconversion rate against WT-SARS-CoV-2

Time point	Anti-spike RBD (BAU/mL) (median, IQR)		P-value	% Inhibition (median, IQR)		P-value	Seroconversion rate (n, %)		P-value
	AZD1222/ mRNA1273 (N=92)*	BNT162b2/ mRNA1273 (N=16)		AZD1222/ mRNA1273 (N=92)*	BNT162b2/ mRNA1273 (N=16)		AZD1222/ mRNA1273 (N=92)*	BNT162b2/ mRNA1273 (N=16)	
Overall									
Before the 3rd dose	48.9 (40.4, 59.0)	49.4 (30.4, 80.3)	0.964	30.3 (17.6, 45.2)	23.4 (10.0, 47.5)	0.324	46 (50.0)	6 (37.5)	0.356
4-week after the 3rd dose	1,227.4 (1,078.8, 1,396.5)	2,437.4 (1,803.2, 3,294.8)	<0.001	98.4 (97.8, 98.5)	98.6 (98.4, 98.7)	0.005	91 (98.9)	16 (100.0)	1.000
Before the 4th dose	249.4 (212.5, 292.7)	369.5 (267.4, 510.6)	0.056	87.5 (64.3, 95.7)	92.6 (81.9, 97.5)	0.102	90 (97.8)	16 (100.0)	1.000
4-week after the 4th dose	5,121.7 (4,640.1, 5,653.3)	3,700.4 (3,001.4, 4,562.2)	0.011	98.2 (98.0, 98.3)	98.2 (98.0, 98.4)	0.305	89 (98.9)	16 (100.0)	1.000
Duration from the 2nd dose ≤ 90 days									
Before the 3rd dose	69.1 (57.2, 83.4)	82.7 (52.9, 129.3)	0.469	32.7 (18.6, 49.9)	39.4 (23.8, 52.6)	0.498	36 (59.0)	6 (60.0)	0.953
4-week after the 3rd dose	1,169.3 (1,004.4, 1,361.3)	2,635.5 (1,619.1, 4,290.0)	0.002	98.4 (97.6, 98.5)	98.6 (98.4, 98.7)	0.004	61 (100.0)	10 (100.0)	-
Before the 4th dose	224.9 (186.5, 271.2)	344.0 (210.9, 561.3)	0.090	86.4 (61.2, 95.7)	91.7 (70.3, 97.5)	0.209	60 (98.4)	10 (100.0)	1.000
4-week after the 4th dose	5,097.7 (4,496.4, 5,779.3)	4,311.6 (3,197.9, 5,813.1)	0.308	98.2 (97.9, 98.3)	98.3 (98.1, 98.4)	0.075	60 (100.0)	10 (100.0)	-
Duration from the 2nd dose > 90 days									
Before the 3rd dose	24.7 (18.1, 33.7)	20.9 (11.0, 40.0)	0.653	21.7 (17.2, 35.7)	4.6 (1.1, 12.6)	0.002	10 (32.3)	0 (0.0)	0.162
4-week after the 3rd dose	1,350.3 (1,053.3, 1,731.2)	2,139.9 (1,555.9, 2,943.0)	0.114	98.4 (98.1, 98.5)	98.5 (98.3, 98.7)	0.364	30 (96.8)	6 (100.0)	1.000
Before the 4th dose	305.6 (225.9, 413.5)	416.2 (250.2, 692.4)	0.384	88.5 (70.5, 95.7)	94.0 (86.8, 97.5)	0.342	30 (96.8)	6 (100.0)	1.000
4-week after	5,170.1	2,868.2	0.004	98.2	98.0	0.496	29	6	1.000

the 4th dose	(4,371.4, 6,114.8)	(2,434.3, 3,379.4)		(98.1, 98.3)	(97.7, 98.4)		(96.7)	(100.0)	

*N=90 for participants at 4 weeks after the fourth dose
