# Supplementary material for: Safety and immunogenicity of the third and fourth doses of vaccine against SARS-CoV-2 following a 2-dose regimen of inactivated whole-virion SARS-CoV-2 vaccine
Source: Sci Rep. 2023 Nov 13;13:19736. doi: 10.1038/s41598-023-45735-7 (PMC10643552; doi:10.1038/s41598-023-45735-7)
Supplement: Supplementary file 2 — Supplementary Table 2. [file 41598_2023_45735_MOESM2_ESM.pdf]

Table S2. Percent inhibition and seroconversion rate against SARS-CoV-2 variant of concerns

| Time point | % Inhibition (median, IQR) | | P-value | Seroconversion rate (n, %) | | P-value |
|---|---|---|---|---|---|---|
| | AZD1222/ mRNA1273 (N=92)* | BNT162b2/ mRNA1273 (N=16) | | AZD1222/ mRNA1273 (N=92)* | BNT162b2/ mRNA1273 (N=16) | |
| B.1.1.7 [Alpha] | | | | | | |
| 4-week after the 3rd dose | 97.0 (94.1, 98.1) | 96.2 (95.5, 96.7) | 0.097 | 90 (97.8) | 16 (100.0) | 1.000 |
| Before the 4th dose | 84.9 (65.7, 93.3) | 82.7 (68.0, 96.0) | 0.551 | 87 (94.6) | 16 (100.0) | 1.000 |
| 4-week after the 4th dose | 98.5 (98.2, 98.6) | 98.4 (98.1, 98.5) | 0.136 | 89 (98.9) | 16 (100.0) | 1.000 |
| B.1.351 [Beta] | | | | | | |
| 4-week after the 3rd dose | 91.8 (81.9, 95.8) | 93.3 (91.6, 94.3) | 0.762 | 88 (95.7) | 16 (100.0) | 1.000 |
| Before the 4th dose | 68.3 (41.6, 78.6) | 74.9 (48.0, 89.9) | 0.169 | 80 (87.0) | 16 (100.0) | 0.207 |
| 4-week after the 4th dose | 98.2 (97.7, 98.4) | 97.6 (97.0, 97.9) | 0.002 | 86 (95.6) | 16 (100.0) | 1.000 |
| B.1.617.2 [Delta] | | | | | | |
| 4-week after the 3rd dose | 97.9 (96.8, 98.3) | 93.5 (91.7, 93.8) | <0.001 | 90 (97.8) | 15 (93.8) | 0.385 |
| Before the 4th dose | 84.4 (61.8, 93.3) | 74.1 (55.9, 92.7) | 0.716 | 90 (97.8) | 16 (100.0) | 1.000 |
| 4-week after the 4th dose | 98.5 (98.2, 98.6) | 98.1 (97.8, 98.5) | 0.085 | 86 (95.6) | 16 (100.0) | 1.000 |
| B.1.1.529 [Omicron] | | | | | | |
| 4-week after the 3rd dose | 62.9 (33.6, 79.6) | 80.5 (62.7, 87.7) | 0.046 | 70 (76.1) | 13 (81.3) | 0.759 |
| Before the 4th dose | 12.7 (3.5, 28.8) | 8.5 (5.6, 25.3) | 0.795 | 20 (21.7) | 3 (18.8) | 1.000 |
| 4-week after the 4th dose | 92.9 (77.6, 96.3) | 81.2 (72.9, 87.2) | 0.035 | 84 (93.3) | 15 (93.8) | 1.000 |

*N=90 for participants at 4 weeks after the fourth dose
