# Supplementary material for: Safety and immunogenicity of the third and fourth doses of vaccine against SARS-CoV-2 following a 2-dose regimen of inactivated whole-virion SARS-CoV-2 vaccine
Source: Sci Rep. 2023 Nov 13;13:19736. doi: 10.1038/s41598-023-45735-7 (PMC10643552; doi:10.1038/s41598-023-45735-7)
Supplement: Supplementary file 3 — Supplementary Table 3. [file 41598_2023_45735_MOESM3_ESM.pdf]

Table S3. RBD-specific antibodies, % inhibition, and seroconversion rate against WT-SARS-CoV-2

Time point	Anti-spike RBD (BAU/mL) (median, IQR)		P-value	% Inhibition (median, IQR)		P-value	Seroconversion rate (n, %)		P-value
	BNT162b2/ BNT162b2 (N=76)*	BNT162b2/ mRNA1273 (N=16)		BNT162b2/ BNT162b2 (N=76)*	BNT162b2/ mRNA1273 (N=16)		BNT162b2/ BNT162b2 (N=76)*	BNT162b2/ mRNA1273 (N=16)	
Overall									
Before the 3rd dose	43.5 (36.1, 52.3)	49.4 (30.4, 80.3)	0.557	20.8 (10.7, 31.0)	23.4 (10.0, 47.5)	0.647	21 (27.6)	6 (37.5)	0.547
4-week after the 3rd dose	2,973.1 (2,596.3, 3,404.5)	2,437.4 (1,803.2, 3,294.8)	0.223	98.5 (98.4, 98.6)	98.6 (98.4, 98.7)	0.642	76 (100.0)	16 (100.0)	-
Before the 4th dose	364.6 (304.8, 436.2)	365.5 (312.7, 427.2)	0.950	94.3 (85.1, 98.1)	92.6 (81.9, 97.5)	0.487	75 (98.7)	16 (100.0)	1.000
4-week after the 4th dose	2,679.3 (2,362.5, 3,038.7)	3,700.4 (3,001.4, 4,562.2)	0.028	98.2 (97.9, 98.3)	98.2 (98.0, 98.4)	0.514	75 (100.0)	16 (100.0)	-
Duration from the 2nd dose ≤ 90 days									
Before the 3rd dose	63.8 (54.8, 74.4)	82.7 (52.9, 129.3)	0.174	26.8 (17.7, 33.7)	39.4 (23.8, 52.6)	0.093	18 (38.3)	6 (60.0)	0.294
4-week after the 3rd dose	2,567.6 (2,181.1, 3,022.5)	2,635.5 (1,619.1, 4,290.0)	0.897	98.5 (98.4, 98.6)	98.6 (98.4, 98.7)	0.274	47 (100.0)	10 (100.0)	-
Before the 4th dose	291.9 (243.2, 350.4)	344.0 (210.9, 561.3)	0.459	91.9 (83.0, 96.6)	91.7 (70.3, 97.5)	1.000	46 (97.9)	10 (100.0)	1.000
4-week after the 4th dose	2,617.9 (2,197.7, 3,118.5)	4,311.6 (3,197.9, 5,813.1)	0.015	98.1 (97.9, 98.3)	98.3 (98.1, 98.4)	0.145	47 (100.0)	10 (100.0)	-
Duration from the 2nd dose > 90 days									
Before the 3rd dose	23.4 (17.1, 31.8)	20.9 (11.0, 40.0)	0.758	12.7 (3.0, 19.3)	4.6 (1.1, 12.6)	0.201	3 (10.3)	0 (0.0)	1.000
4-week after the 3rd dose	3,770.6 (3,020.4, 4,707.0)	2,139.9 (1,555.9, 2,943.0)	0.028	98.6 (98.5, 98.6)	98.5 (98.3, 98.7)	0.538	29 (100.0)	6 (100.0)	-
Before the 4th dose	523.0 (372.5, 734.3)	416.2 (250.2, 692.4)	0.550	97.8 (92.1, 98.2)	94.0 (86.8, 97.5)	0.168	29 (100.0)	6 (100.0)	-
4-week after the 4th dose	2,785.7 (2,329.8, 3,330.7)	2,868.2 (2,434.3, 3,379.4)	0.880	98.2 (98.0, 98.3)	98.0 (97.7, 98.4)	0.442	28 (100.0)	6 (100.0)	-

*N=75 for participants at 4 weeks after the fourth dose
