# Supplementary material for: Safety and immunogenicity of the third and fourth doses of vaccine against SARS-CoV-2 following a 2-dose regimen of inactivated whole-virion SARS-CoV-2 vaccine
Source: Sci Rep. 2023 Nov 13;13:19736. doi: 10.1038/s41598-023-45735-7 (PMC10643552; doi:10.1038/s41598-023-45735-7)
Supplement: Supplementary file 4 — Supplementary Table 4. [file 41598_2023_45735_MOESM4_ESM.pdf]

Table S4. Percent Inhibition and seroconversion rate against SARS-CoV-2 variant of concerns

Time point	% Inhibition (median, IQR)		P-value	Seroconversion rate (n, %)		P-value
	BNT162b2/ BNT162b2 (N=76)*	BNT162b2/ mRNA1273 (N=16)		BNT162b2/ BNT162b2 (N=76)*	BNT162b2/ mRNA1273 (N=16)	
B.1.1.7 [Alpha]						
4-week after the 3rd dose	96.5 (96.2, 96.7)	96.2 (95.5, 96.7)	0.117	75 (98.7)	16 (100.0)	1.000
Before the 4th dose	86.6 (71.6, 97.2)	82.7 (68.0, 96.0)	0.639	73 (96.1)	16 (100.0)	1.000
4-week after the 4th dose	98.3 (98.1, 98.5)	98.4 (98.1, 98.5)	0.743	75 (100.0)	16 (100.0)	-
B.1.351 [Beta]						
4-week after the 3rd dose	93.8 (92.6, 94.5)	93.3 (91.6, 94.3)	0.356	75 (98.7)	16 (100.0)	1.000
Before the 4th dose	74.4 (58.5, 90.7)	74.9 (48.0, 89.9)	0.753	73 (96.1)	16 (100.0)	1.000
4-week after the 4th dose	97.6 (96.5, 98.0)	97.6 (97.0, 97.9)	0.958	75 (100.0)	16 (100.0)	-
B.1.617.2 [Delta]						
4-week after the 3rd dose	93.8 (92.8, 94.3)	93.5 (91.7, 93.8)	0.089	75 (98.7)	15 (93.8)	0.319
Before the 4th dose	81.6 (63.0, 96.3)	74.1 (55.9, 92.7)	0.365	73 (96.1)	16 (100.0)	1.000
4-week after the 4th dose	98.0 (97.7, 98.4)	98.1 (97.8, 98.5)	0.528	75 (100.0)	16 (100.0)	-
B.1.1.529 [Omicron]						
4-week after the 3rd dose	81.7 (67.3, 91.9)	80.5 (62.7, 87.7)	0.500	70 (92.1)	13 (81.3)	0.187
Before the 4th dose	17.6 (7.8, 39.1)	8.5 (5.6, 25.3)	0.161	25 (32.9)	3 (18.8)	0.374
4-week after the 4th dose	85.3 (72.7, 92.5)	81.2 (72.9, 87.2)	0.370	69 (92.0)	15 (93.8)	1.000

*N=90 for participants at 4 weeks after the fourth dose
